# The impact of early-life exposures on growth and adult gut microbiome composition is dependent on genetic strain and parent- of- origin

**DOI:** 10.1186/s40168-025-02130-w

**Published:** 2025-06-16

**Authors:** M. Nazmul Huda, Emer Kelly, Keri Barron, Jing Xue, William Valdar, Lisa M. Tarantino, Sarah Schoenrock, Folami Y. Ideraabdullah, Brian J. Bennett

**Affiliations:** 1 Department of Nutrition, University of California Davis, Davis, CA USA; 2 Obesity and Metabolism Research Unit, Western Human Nutrition Research Center, ARS, USDA, Davis, CA 95616 USA; 3 Human Nutrition, School of Agriculture and Food Science, University College Dublin, Dublin, Ireland; 4 Department of Genetics, School of Medicine, University of North Carolina at Chapel Hill, Chapel Hill, NC USA; 5 Nutrition Research Institute, University of North Carolina at Chapel Hill, Kannapolis, NC USA; 6 Department of Nutrition, Gillings School of Public Health, University of North Carolina at Chapel Hill, Chapel Hill, NC USA

**Keywords:** Gut microbiota, Genetics, Developmental environment, Antibiotics, Parent-of-origin

## Abstract

**Background:**

Early-life exposure to environmental factors can have long-lasting impacts on offspring health into adulthood and therefore is an emerging public health concern. In particular, the impact of maternal environmental exposures such as diet and antibiotic use on the establishment of the offspring gut microbiome has been recently highlighted as a potential link to disease risk. However, the long-term effects are poorly understood. Moreover, interindividual host genetic differences have also been implicated in modulating the gut microbiome, suggesting that these differences may modulate susceptibility to environmentally induced dysbiosis and exacerbate related health outcomes. Our understanding of how the developmental environment and genetics interact to modulate offspring long-term gut microbiota and health is still limited.

**Methods:**

In this study, we investigated the effects of early exposure to known or putative dietary insults on the microbiome (antibiotic exposure, protein deficiency, and vitamin D deficiency) in a novel population of mice. Dams were maintained on purified AIN93G antibiotic-containing (AC), low-protein (LP), low-vitamin D (LVD), or mouse control (CON) diets from 5 weeks prior to pregnancy until the end of lactation. After weaning, mice were transferred to new cages and fed a standardized chow diet. The parent-of-origin (PO) effect was determined via F1 offspring from reciprocal crosses of recombinant inbred intercross (RIX) of Collaborative Cross (CC) mice, where all F1 offspring within a reciprocal pair were genetically identical except for the *X*- and *Y*-chromosomes and mitochondrial genomes. We assayed offspring bodyweight and the gut bacterial microbiota via 16S rRNA gene sequencing at 8 weeks of age.

**Results:**

Our study revealed that early developmental exposure to antibiotics, protein deficiency, and vitamin D deficiency had long-lasting effects on offspring bodyweight and gut microbial diversity and composition, depending on the genetic background. Several bacterial genera and ASVs, including *Bacteroides*, Muribaculaceae, *Akkermansia*, and *Bifidobacterium*, are influenced by developmental insults. We also observed a significant effect of PO on offspring gut microbiota and growth. For example, the offspring of CC011xCC001 mice had increased bodyweight, microbial diversity indices, and several differential bacterial abundances, including those of *Faecalibaculum*, compared with those of the corresponding reciprocal cross CC001xCC011.

**Conclusion:**

Our results show that maternal exposure to nutritional deficiencies and antibiotics during gestation and lactation has a lasting impact on offspring gut microbiota composition. The specific responses to a diet or antibiotic can vary among F1 strains and may be driven by maternal genetics.

Video Abstract

**Supplementary Information:**

The online version contains supplementary material available at 10.1186/s40168-025-02130-w.

## Introduction

Early-life exposure can have long-lasting effects on the health of progeny [1, 2]. The development of an infant microbiota initiates prior to birth and is heavily influenced by maternal factors [3]. Mothers’ nutritional status, use of medication, genetics, and microbiota are important early-life exposures for newborns, and these maternal factors play critical roles in the colonization of a newborn’s gut microbiota [4]. However, the long-term impact of these changes remains unclear.

Macro- and micronutrient deficiencies are common among pregnant and lactating women around the world and have clear public health impacts. For example, protein deficiency among pregnant and lactating women, a common problem in developing countries, is linked to various communicable and noncommunicable diseases [5, 6]. Some effects of protein deficiency in children can be mediated through dysbiosis [7]. Similarly, maternal vitamin D deficiency, a global public health issue [8–11], is associated with various diseases and an altered gut microbiota in infants [12, 13]. Although a few studies have evaluated the effects of maternal protein and vitamin D deficiency on an infant’s gut microbiota, none has examined the long-term effects during adulthood. Likewise, maternal and neonatal antibiotic therapies are commonly used [14] and affect the early colonization of gut microbiota in infants [15, 16]. Even brief exposure to antibiotics during the neonatal period has a large effect on the gut microbiota and subsequent health [1]. Using mouse models, we previously showed that antibiotic exposure alone during gestation and lactation altered pup survival and offspring growth from birth into adulthood [17]. However, our understanding of the effects of antibiotic use, protein deficiency, and vitamin D deficiency during the developmental period on the gut microbiota and health is very limited [15, 18].

Moreover, the effects of developmental insults on the microbiome and health of offspring may be modulated by genetics [19]. Host physiology and genetics regulate the gut microbiota through a variety of mechanisms, including variations in bile acid metabolism [20], mucosal gut structure [21], and antimicrobial peptides and secretory immunoglobulin A [22]. Additionally, whether offspring inherit causal genotypes from the mother vs. the father (parent-of-origin, PO) has a substantial impact on growth and development [23]. It is plausible but unknown if PO has long-lasting effects on the microbiota of offspring and what the physiological effects of PO-mediated microbiota compositional changes could be.

In this study, we used Collaborative Cross (CC) mice to generate reciprocal F1 progeny. We altered early environmental exposures by feeding the dams experimental purified diets, including antibiotic-containing (AC), low protein (LP), low vitamin D (LVD), or control diets, from 5 weeks before pregnancy until the end of lactation. CC mice are recombinant inbred lines generated from eight founder strains consisting of three major *Mus musculus* subspecies: *domesticus* (A/J, C57BL/6 J, 129S1/SvImJ, NOD/ShiLtJ, NZO/HlLtJ, WSB/EiJ), *castaneus* (CAST/EiJ), and *musculus* (PWK/PhJ) [24]. CC mice were designed specifically for the analysis of gene × environment effects on complex phenotypes [25]. We take advantage of this powerful CC mouse population to detect how early environmental exposures, and interactions between genetic background and PO modulate an offspring gut microbiota into adulthood and whether these changes coincide with detectable effects on offspring bodyweight.

## Methods

### Study design and sample collection

**Fig. 1:**
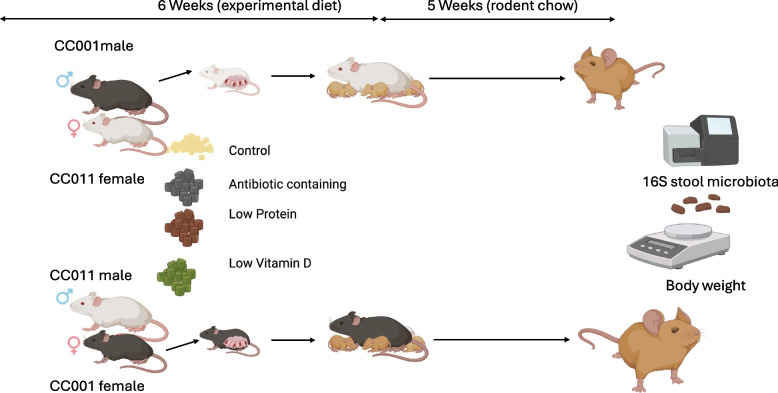
Study design. Inbred CC001, CC011, CC004, CC017, CC041, and CC051 male and female mice were obtained to generate male offspring (F1) by reciprocal crossbreeding. Mothers were placed on experimental diets (AC, LP, LVD, and control) 5 weeks before mating, throughout gestation, and until offspring weaning. F1 male offspring were weaned on postnatal day 21 and transferred to standard rodent chow. At 8 weeks of age, F1 mice were weighed and euthanized to collect cecum samples. The cecum was flash-frozen and stored at − 80 °C until analysis

We conducted three separate experiments with a common control group to understand the effects of maternal antibiotic use (*Experiment 1*), protein deficiency (*Experiment 2*), and vitamin D deficiency (*Experiment 3*) during the developmental period on offspring gut microbiota composition during adulthood using Collaborative Cross (CC) mice (Fig. 1). Inbred CC001/Unc, CC011/Unc, CC004/TauUnc, CC017/Unc, CC041/TauUnc, and CC051/TauUnc (further referred to without the suffix) mice were obtained from the UNC Systems Genetics Core Facility (Chapel Hill, NC, USA) between 2013 and 2015. Male offspring (F1) were generated from CC crosses by trio or harem breeding (dam × sire, CC001xCC011, and reciprocal cross CC011xCC001). Therefore, the F1 offspring from a pair of reciprocal crosses are genetically identical except for the sex chromosomes and mitochondrial DNA. The mothers were placed on diets beginning 5 weeks before mating, throughout gestation, and until the offspring were weaned. The treatment diets included antibiotic-containing (AC), low-protein (LP), and low-vitamin D (LVD) diets or AIN-93G as a control diet (CON). Each diet group had 36–46 (AC, *n* = 36; LP, *n* = 49; LVD, *n* = 43; and CON, *n* = 46) F1 mice. This treatment window ensures the effect of diet before conception and throughout the early development of the offspring. The vivarium temperature was maintained between 21 and 23 °C with a 12-h light cycle. Sterilized water and rodent chow were provided ad libitum. F1 male offspring were weaned at postnatal day 21 and matured to adulthood. All dams in this study were euthanized at pup weaning, and offspring were transferred to standard rodent chow (Teklad 8604, Harlan Laboratories). At 8 weeks of age, biological samples were collected, and the F1 mice were euthanized. The cecum was flash-frozen and stored at − 80 °C until analysis. Animal handling followed the Guide for the Care and Use of Laboratory Animals under the corresponding animal use protocol at the University of North Carolina at Chapel Hill.

### Diet composition

Custom diets were obtained from Dyets (PA, USA). The dams were fed AIN-93G rodent chow (no. 110700) (Dyets Inc., PA, USA) as the control or one of the three intervention diets (AC — no. 518893, LP — no. 102787, and LVD — no. 119266) from 5 weeks before mating to the weaning of the pups. The detailed composition of the diet is available in Supplemental Table 1. The AC diet was a modified AIN-93G with 1% succinyl sulfathiazole supplemented with 14.48 g/kg choline bitartrate. The LVD diet was a modified AIN-93G diet containing no vitamin D (0 IU/kg), and the LP diet was a modified AIN-93G diet with only 7.5% protein (approximately 60% reduced protein levels compared with those of the control AIN-93G). After weaning, the pups were transferred to a standard rodent chow diet (Teklad 8604, Harlan Laboratories).

### Microbiota assay

Total DNA from pup cecum was extracted using the Maxwell 16 tissue DNA Purification kit (Promega, WI, USA). The 16S rRNA gene V4 region was amplified using the Earth Microbiome Project 16S Illumina amplicon protocol with a 515 forward and 806 barcoded reverse primer set [26, 27]. The amplicon PCR product was cleaned using the Wizard SV 96 PCR Clean-Up System (Promega, WI, USA). The final DNA concentration was quantified using a Quant-iT PicoGreen dsDNA assay (Thermo Fisher, MA, USA), and an equimolar mixture was made. The multiplexed amplicon was sequenced using a MiSeq Illumina platform with 2 × 250-bp paired-end sequencing. The obtained sequence data were demultiplexed and analyzed using the QIIME2 pipeline [28]. The ASV table was generated using DADA-2 [29]. Taxonomy was assigned using the SILVA 138 reference database [30] customized for the 16S V4 (515 F/806R) region of sequences at the threshold of 99% pairwise identity.

### Statistical analysis

Statistical analyses were performed using R version v4.1.1 for Windows [31]. Data were analyzed for normality via the Shapiro‒Wilk test and a QQ-normal plot. A Shapiro‒Wilk *W*-value ≥ 0.95 was considered normal. Nonnormal metadata variables were transformed by natural log, square root, square, or Box–Cox power transformation. If no appropriate transformation was found, the variables were rank transformed. The Shannon diversity *α*-rarefaction plot plateaued at 5000 sequences. The ASV table was rarified at 24,000 sequences per sample. Differences in microbial community *β*-diversity (weighted UniFrac, unweighted UniFrac, and Bray Curtis) were tested using the ADONIS function (permutational multivariate analysis of variance — PERMANOVA [32]) in the R package vegan [33]. Principal coordinate analysis (PCoA) was carried out via phyloseq [34]. Differential microbiota abundance was analyzed using ANCOM-2 [35]. ANCOM-2 models were FDR (BH method) corrected. The feature table was normalized using CLR for ANCOM-2 analysis. Comparisons between multiple phenotypic groups were carried out by ANCOVA while adjusting for confounding factors (e.g., diet or cross). Associations between phenotypic groups and bodyweight were accomplished using MaAsLin2 [36]. The Wilcoxon rank-sum test was used for comparisons between crosses in a RIX. All *P*-values reported in the study were from two-tailed tests. *P*-values were corrected for multiple comparisons using the Benjamini-Hochberg (BH) procedure. *P*-values < 0.05 or BH-adjusted *P*-values (*q*-value) < 0.05 were considered significant. Graphs were prepared using ggplot2 [37].

## Results

### Early-life antibiotic exposure has a long-term effect on growth and the gut microbiota

In this study, we investigated the effects of early-life (defined as gestation through weaning) exposure to antibiotics, protein deficiency, and vitamin D deficiency on offspring bodyweight and the gut bacterial microbiota later at 8 weeks of age (Fig. 1). The overall distribution of microbial abundance among the treatment diets and crosses is depicted in Supplemental Fig. 1.

**Fig. 2:**
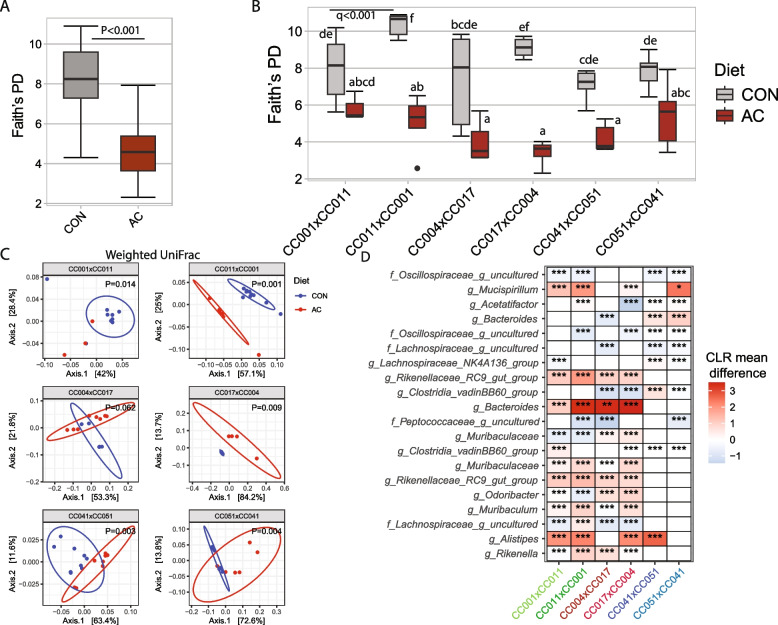
Antibiotic exposure during development affects bodyweight and the gut microbiota at 8 weeks of age. **A** Faith’s phylogenetic diversity score of offspring gut microbiota at 8 weeks in AC and control groups. ANCOVA models were adjusted for the cross. **B** Comparisons of Faith’s PD between reciprocal crosses within each diet. The Wilcoxon test was used to compare each pair of reciprocal crosses. *P*-values were corrected for multiple comparisons using the Benjamini–Hochberg method. **C** Weighted UniFrac *β*-diversity principal coordinate plot of the offspring gut microbiota stratified by crosses. Colors represent maternal AC or CON diets as indicated. The ellipse indicates a 95% CI of the clusters by maternal diets. **D** Heatmap of differential ASV abundance in the gut microbiota of offspring whose mother maintained on the AC diet compared to the control group. The top 20 most differentially abundant (based on cumulative ANCOM *W*-value) ASVs were selected for the graph. On the *Y*-axis, maximum taxonomic information has been presented. Colors represent CLR mean differences (effect size) of the ASV abundance between AC and CON diets. Red indicates higher, and blue represents lower ASV abundance in the AC diet compared to the mouse control diet. White represents a nonsignificant result obtained from ANCOM analysis. ANCOM models were FDR (BH method) corrected, and a significant sub-hypothesis test at a level of *adj.P* < 0.05 was counted towards the *W*-value. “***” = ≥ *W*_0.9_, “**” = ≥ *W*_0.8_, “*” = ≥ *W*_0.7_, “.” = ≥ *W*_0.6_. The full list of the differential ASV abundance is available in Supplemental Table 6. Corresponding differential genera abundance has been depicted in Supplemental Table 7

In Experiment 1, we sought to understand the long-term effects of maternal antibiotic exposure during the developmental period on offspring growth and the gut microbiota in adulthood. Female mice were fed an antibiotic-containing diet (AC) or a control diet 5 weeks before mating until weaning (“Methods”). We subsequently quantified offspring gut microbiota at 8 weeks of age. Offspring mice were derived from three pairs of reciprocal crosses of CC mice, which additionally provided a unique opportunity to identify both genetic and PO effects on offspring growth and gut microbiota. We wanted to understand whether early-life antibiotic exposure had long-lasting effects on the gut microbiota of offspring at 8 weeks of age. We first determined if the microbial *α*-diversity differed between the AC and control diet groups. Microbial Faith’s PD, Shannon diversity index, and observed species *α*-diversity measures all were significantly (*P* < 0.0001) lower in the antibiotic-fed mice compared to the control-fed mice, independent of the genetic background of the mice (Fig. 2A and Supplemental Tables 2, 3, and 4). We also observed a significant diet × cross interaction effect on the *α*-diversity measures (*P* < 0.001). Therefore, we further determined the effect of diet on microbial *α*-diversity stratified by cross. We found that AC treatment led to significantly lower Faith’s PD for offspring from the CC011xCC001, CC004xCC017, and CC017xCC004 crosses, whereas the other three genetic lineages were unaffected (Fig. 2B and Supplemental Table 2). Additionally, compared with the corresponding control diet groups, the AC-treated CC041xCC051 and CC051xCC041 groups had a lower Shannon diversity index (Supplemental Table 3). AC-treated CC051xCC041 also had a lower observed species (Supplemental Table 4). Statistical analysis of *α*-diversity between crosses within a RIX and diet pairs indicated that there were consistent effects within RIX strains, with one exception. A significantly (*q* < 0.001) higher Faith’s PD was observed in the CC011xCC001 offspring than in the CC001xCC011 offspring fed the control diet (Fig. 2B). Next, we determined the effect of early-life antibiotic use on gut microbial *β*-diversity using ADONIS. We found that diet (AC vs. CON) explained 8.8% (weighted UniFrac, *P* = 0.001) to 14.5% (unweighted UniFrac, *P* = 001) of the variability in the microbiota independent of genetic background (Table 1). A significant (*P* = 0.001) interaction between diet and cross was observed for all three *β*-diversity measures, indicating that the effect of maternal antibiotic use on the microbial composition of offspring varied depending on the genetic background (Table 1, Fig. 2C, and Supplemental Fig. 2). We further determined if PO modulates the association between antibiotic insult and microbial *β*-diversity. To do so, we performed ADONIS analysis on crosses in a RIX pair within each diet (e.g., CC001xCC011 and CC011xCC001 offspring in the AC group). Our analysis showed that the microbial *β*-diversity was significantly modulated by PO, which explained approximately 20 to 50% of the variability, depending on the genetic background (Supplemental Table 5). We also found that PO had a significant effect on offspring *β*-diversity in the control group, which explained approximately 20 to 40% of the variability in the microbiota (Supplemental Table 5). These results indicate that early-life exposure to antibiotics has a long-lasting effect on the gut microbial composition, which is modulated by the host genetic background in a PO-dependent manner.

**Table 1:** Association of microbial *β*-diversity with the developmental environment, cross, and their interactions

	**Developmental environment**		**Cross**		**Developmental environment: cross**	
	**R2**	***P***	**R2**	**P**	**R2**	***P***
Weighted UniFrac						
LVD vs. CON	0.013	0.149	0.286	0.001	0.074	0.020
LP vs. CON	0.015	0.056	0.248	0.001	0.116	0.001
AC vs. CON	0.088	0.001	0.158	0.001	0.239	0.001
Unweighted UniFrac						
LVD vs. CON	0.015	0.028	0.272	0.001	0.093	0.001
LP vs. CON	0.020	0.002	0.259	0.001	0.096	0.001
AC vs. CON	0.145	0.001	0.176	0.001	0.145	0.001
Bray–Curtis						
LVD vs. CON	0.019	0.007	0.285	0.001	0.099	0.001
LP vs. CON	0.020	0.001	0.269	0.001	0.105	0.001
AC vs. CON	0.093	0.001	0.174	0.001	0.171	0.001

We further determined the differential ASVs and genus abundances in the offspring gut microbiota between the AC and CON groups. Since the interaction between diet and cross on microbial *α*-diversity and *β*-diversity was significant, we performed differential ASV abundance between AC and control stratified by the cross (Supplemental Fig. 4). Compared with the control group, the AC group presented significantly different abundances of several ASVs (Fig. 2D and Supplemental Table 6) and genera (Supplemental Table 7). For example, the abundances of *Bacteroides* at the ASV level (Supplemental Table 6) and the genus level (Supplemental Table 7) were reduced more than threefold in offspring from all crosses fed the AC diet compared with those fed the control diet. Similarly, *Muribaculaceae* increased approximately threefold in CC017xCC004, CC011xCC001, and CC041xCC051 offspring from the AC group compared with their corresponding controls. In contrast, *Akkermansia* decreased threefold in CC051xCC041 offspring in the AC group compared with those in the control group.

### Protein deficiency during the developmental period has a long-lasting effect on the microbiota and growth

**Fig. 3:**
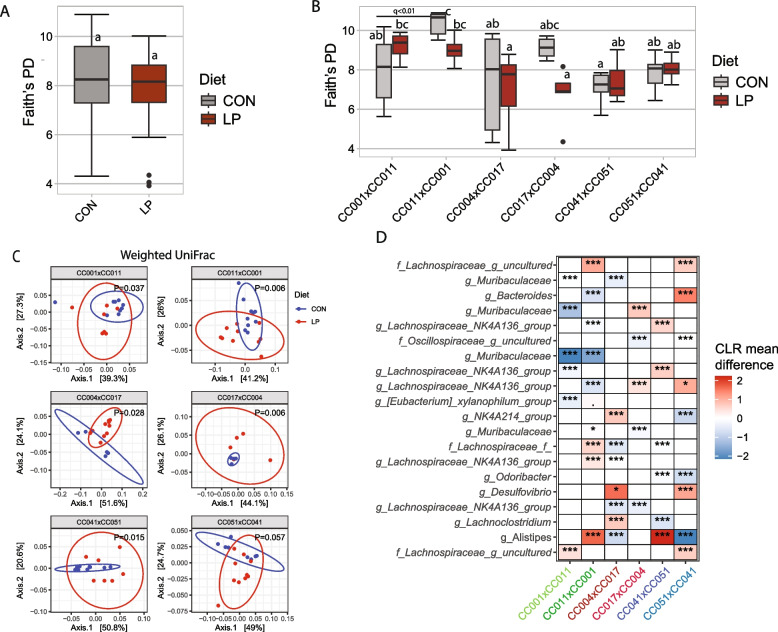
Protein deficiency during the developmental period affects bodyweight and the gut microbiota at 8 weeks of age. **A** Faith’s phylogenetic diversity score of offspring gut microbiota at 8 weeks in LP and control groups. ANCOVA models were adjusted for the cross. **B** Comparisons of Faith’s PD between reciprocal crosses within each diet. The Wilcoxon test was used to compare each pair of reciprocal crosses. *P*-values were corrected for multiple comparisons using the Benjamini–Hochberg method. **C** Weighted UniFrac *β*-diversity principal coordinate plot of the offspring gut microbiota stratified by crosses. Colors represent different maternal LP or CON diets, as indicated. The ellipse indicates a 95% CI of the clusters by maternal diets. **D** Heatmap of differential ASV abundance in the gut microbiota of offspring mother maintained on an LP diet, compared to the control group. The top 20 most differentially abundant (based on cumulative ANCOM *W*-value) ASV were selected for the graph. On the *Y*-axis, maximum taxonomic information has been presented. Color represents CLR mean differences (effect size) of ASV abundance between LP and CON diets. Red indicates higher, and blue represents lower ASV abundance in the LP diet compared to the mouse control diet. White represents a nonsignificant result obtained from ANCOM analysis. ANCOM models were FDR (BH method) corrected, and a significant sub-hypothesis test at a level of *adj.P* < 0.05 was counted towards *W*-value. “***” = ≥ *W*_0.9_, “**” = ≥ *W*_0.8_, “*” = ≥ *W*_0.7_, “.” = ≥ *W*_0.6_. The full list of the differential ASV abundance is available in Supplemental Table 6. Corresponding differential genera abundance has been depicted in Supplemental Table 7

In Experiment 2, we determined the effect of protein deficiency during the developmental period on the gut microbiota at 8 weeks of age. We did not observe any overall significant differences in Faith’s PD, Shannon diversity index, or observed species between the gut microbiota in the control and LP groups (Fig. 3A and Supplemental Tables 2, 3, and 4); however, we observed a significant interaction (*P* < 0.01) between diet and cross on microbial *α*-diversity. Further analysis indicated that CC001xCC011 had a significantly lower Faith’s PD in the LP diet compared to CC001xCC011 (Fig. 3B and Supplemental Table 2). A similar effect of the LP diet was also observed for the Shannon diversity index and observed species (Supplemental Table 3 and Supplemental Table 4).

The effect of the LP diet on the offspring gut microbial *β*-diversity was subsequently assessed (Table 1 and Supplemental Fig. 2). The maternal LP diet had a significant effect on offspring both unweighted UniFrac (*P* = 0.002) and Bray‒Curtis (*P* = 0.001) *β*-diversity measures. The maternal LP diet also affected weighted UniFrac *β*-diversity but did not reach a significant level after adjusting for the cross as a confounding factor. Additionally, we observed a significant (*P* = 0.001) interaction between diet and cross on *β*-diversity, demonstrating that the effect of protein deficiency during the developmental period on offspring gut microbiota was dependent on genetic background (Fig. 3C and Table 2). Additional analysis of *β*-diversity within RIX pairs within a diet (e.g., CC001xCC011 vs CC011xCC001 offspring fed an LP diet) revealed that CC001xCC011 and CC041xCC051 had significantly different *β*-diversity compared to their corresponding RIX. CC011xCC001 and CC051xCC041 in the LP diet, which explained 14–20% of the variability in the microbiota depending on the diversity measures and reciprocal cross pairs (Supplemental Table 5).

**Table 2:** Comparisons of bodyweight between reciprocally crossed CC mice maintained on different diets

Cross	Developmental environment	n	Mean ± SD	Median (25 th, 75 th)	Wilcox *p*-values	Wilcox adj.P
CC001xCC011	CON	9	24.8 ± 1.9	24.9 (23.4, 26.3)	0.00026	0.0031
CC011xCC001	CON	10	29.6 ± 2.18	29.3 (28.6, 31.5)		
CC004xCC017	CON	5	24.7 ± 1.68	24.4 (23.7, 24.6)	0.31	0.74
CC017xCC004	CON	5	23.7 ± 0.884	23.5 (23, 24.2)		
CC041xCC051	CON	9	26.8 ± 1.25	27.1 (26.1, 27.6)	0.67	1.00
CC051xCC041	CON	8	26.4 ± 2.14	25.9 (25.4, 27.7)		
CC001xCC011	AC	3	24.6 ± 0.6	24.6 (24.3, 24.9)	0.67	0.67
CC011xCC001	AC	7	24.1 ± 1.78	23.8 (22.7, 25.5)		
CC004xCC017	AC	8	21.1 ± 1.32	21.7 (20, 22)	1.00	1.00
CC017xCC004	AC	4	21.3 ± 0.711	21.1 (21, 21.4)		
CC041xCC051	AC	8	22.3 ± 0.772	22.4 (21.9, 22.9)	0.95	1.00
CC051xCC041	AC	6	22.5 ± 1.16	22.4 (21.8, 23.3)		
CC001xCC011	LP	7	22 ± 0.979	21.5 (21.3, 22.5)	0.0020	0.012
CC011xCC001	LP	10	24.5 ± 1.45	24.9 (23.4, 25.6)		
CC004xCC017	LP	10	21.7 ± 1.37	21.2 (20.8, 22.8)	0.10	0.48
CC017xCC004	LP	5	20.6 ± 1.06	20.3 (20, 20.4)		
CC041xCC051	LP	7	21.8 ± 1.39	21.5 (20.7, 22.9)	0.87	1.00
CC051xCC041	LP	10	21.8 ± 1.68	21.3 (20.6, 22.5)		
CC001xCC011	LVD	9	26.3 ± 2.13	26.2 (24.7, 28)	0.012	0.028
CC011xCC001	LVD	7	29.6 ± 2.29	30.6 (29.4, 30.9)		
CC004xCC017	LVD	5	25.7 ± 2.29	25.5 (23.6, 27.3)	0.032	0.25
CC017xCC004	LVD	5	23.4 ± 0.936	23.1 (23, 23.2)		
CC041xCC051	LVD	9	27 ± 1.18	26.8 (26.4, 27.4)	0.42	0.78
CC051xCC041	LVD	8	28.5 ± 3.79	29.3 (25.5, 30.8)		

Wilcox tests were performed on each pair of the reciprocal cross. *P*-values were adjusted for multiple comparisons using the BH method. Statistical analyses were performed using ANCOVA

We next focused on the differential ASV and genera abundances in the gut microbiota in LP fed mice. Compared with the control group, the LP diet group had several differentially abundant ASVs (Fig. 3D and Supplemental Table 8) and genera (Supplemental Fig. 5 and Supplemental Table 9). For instance, *Bifidobacterium* abundance was over 20% lower in offspring from CC017xCC004 in LP groups compared to the control group (Supplemental Table 9). Additionally, offspring from CC051xCC041 in the LP group had over 25% reduction in *Akkermansia* abundance compared to control. Our results indicate that depending on the genetic background, protein deficiency during the developmental period shapes offspring gut microbiota, which may last at least until 8 weeks of age in mice.

### Vitamin D deficiency during the developmental period affects offspring gut microbiota during adulthood without affecting growth

**Fig. 4:**
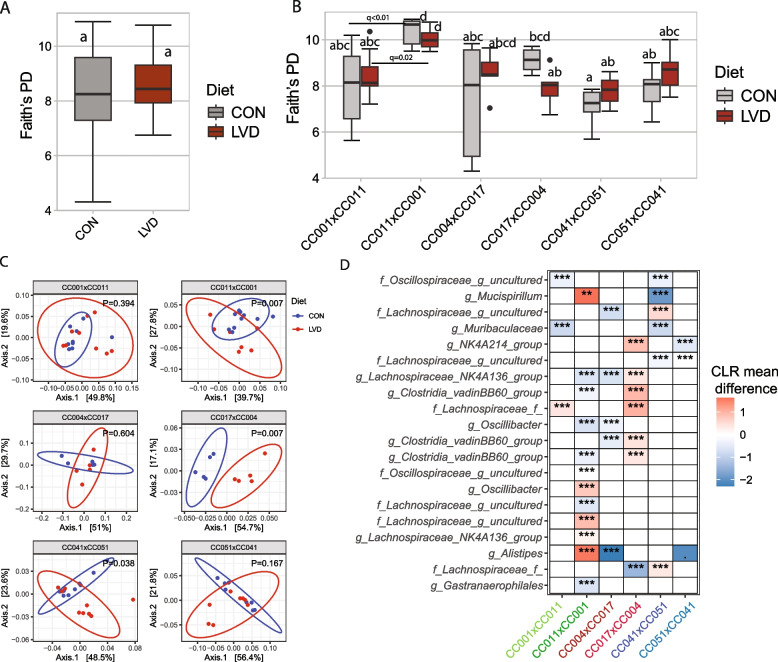
Vitamin D deficiency during the developmental period affects gut microbiota in adulthood but does not affect growth. **A** Faith’s phylogenetic diversity score of offspring gut microbiota at 8 weeks in LVD and control groups. ANCOVA models were adjusted for the cross. **B** Comparisons of Faith’s PD between reciprocal crosses within each diet. The Wilcoxon test was used to compare each pair of reciprocal crosses. *P*-values were corrected for multiple comparisons using the Benjamini–Hochberg method. **C** Weighted UniFrac *β*-diversity principal coordinate plot of the offspring gut microbiota stratified by crosses. Colors represent different maternal LVD or CON diets as indicated. The ellipse indicates a 95% CI of the clusters by maternal diets. **D** Heatmap of differential ASV abundance in the gut microbiota of offspring mother maintained on the LVD diet compared to the control group. The top 20 most differentially abundant (based on cumulative ANCOM W-value) ASV were selected for the graph. On the *Y*-axis, maximum taxonomic information has been presented. Colors represent CLR mean differences (effect size) of the ASC abundance between LVD and CON diets. Red indicates higher, and blue represents lower ASV abundance in the LVD group compared to the control. White represents a nonsignificant result obtained from ANCOM analysis. ANCOM models were FDR (BH method) corrected, and a significant sub-hypothesis test at a level of *adj.P* < 0.05 was counted towards *W*-value. “***” = ≥ *W*_0.9_, “**” = ≥ *W*_0.8_, “*” = ≥ *W*_0.7_, “.” = ≥ *W*_0.6_. The full list of the differential ASV abundance is available in Supplemental Table 6. Corresponding differential genera abundance has been depicted in Supplemental Table 7

In Experiment 3, we determined the consequences of vitamin D deficiency during the developmental period on the gut microbiota at 8 weeks of age. There were no significant differences in microbial *α*-diversity between the LVD and CON groups when all the crosses were combined (Fig. 4A). However, Wilcoxon analysis comparing *α*-diversity between crosses in each RIX pair within the LVD showed several clear PO effects on the microbiota at 8 weeks of age (Fig. 4B and Supplemental Tables 2, 3, and 4). Faith’s PD and observed species *α*-diversity in CC011xCC001 were significantly greater than those in CC001xCC011 (*P* < 0.001, *q* = 0.021) in the LVD group (Fig. 4B and Supplemental Table 2). Moreover, CC041xCC051 offspring in the LVD group had greater Faith’s PD (Supplemental Table 2) and Shannon diversity indices (Supplemental Table 3) than did the corresponding CC051xCC041 offspring but did not significantly differ after adjustment for multiple comparisons.

Although the microbial weighted UniFrac *β*-diversity was not affected by the LVD diet (Table 1), the unweighted UniFrac and Bray‒Curtis *β*-diversity measures were significantly affected by the LVD diet compared with the CON diet (Table 1 and Supplemental Fig. 2). We observed significant diet by cross interaction for all three *β*-diversity measures (Table 2), indicating that the composition of the offspring gut bacteria was affected by LVD depending on the genetic background. We further compared the *β*-diversity of crosses within an RIX pair within the LVD group (e.g., CC001xCC011 vs. CC011xCC001 offspring fed the LVD diet), which revealed that all the crosses had significantly different unweighted UniFrac and Bray‒Curtis *β*-diversity values than did their corresponding RIX values (Supplemental Table 5). This observation indicates that PO has a strong influence on the overall microbial composition of offspring from the LVD group. To understand whether there was any effect of LVD exposure during the developmental period on microbial abundance, we determined the differential bacterial ASV and genus abundance between the LVD and control offspring stratified by crosses. Compared to control, LVD treatment resulted in several differentially abundant ASVs (Fig. 4D and Supplemental Table 10) and genera (Supplemental Table 11), depending on the genetic background. For example, the abundance of *Bifidobacterium* was lower in offspring from CC017xCC004 in the LVD groups than in those from the corresponding control groups (Supplemental Table 11). Similarly, *Rikenella* was depleted in CC051xCC041 offspring in the LVD group compared with those in the control group. Our results demonstrated that exposure to vitamin D deficiency during development may alter several important gut bacteria depending on the genetic background.

### Parent-of-origin modulates the gut microbial composition during adulthood

**Fig. 5:**
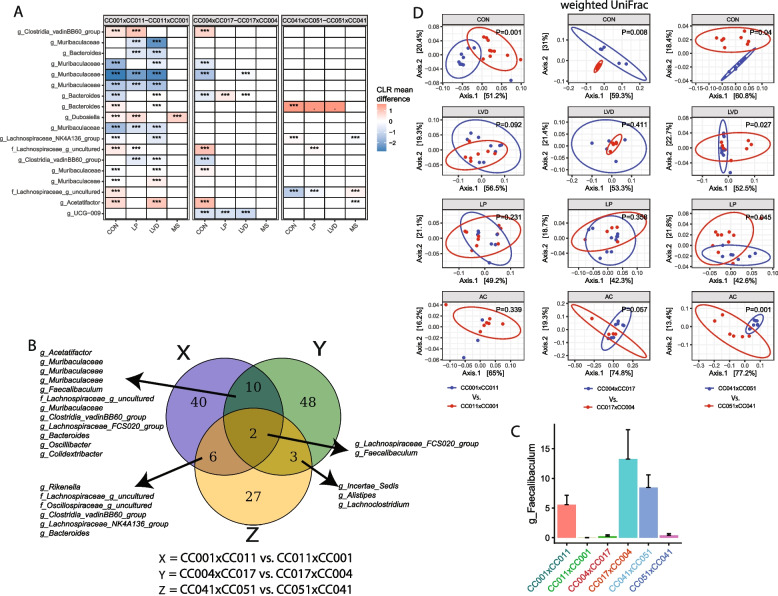
Parent-of-origin is associated with offspring gut microbial *β*-diversity and composition at adulthood. **A** Heatmap of differential ASV abundance between reciprocal crosses as indicated. The top 20 most differentially abundant (based on cumulative ANCOM *W*-value) ASVs were selected for the graph. On the *Y*-axis, maximum taxonomic information has been presented. Colors represent CLR mean differences (effect size) of ASV abundance between corresponding reciprocal crosses (e.g., CC001xCC011 vs CC011xCC001). ANCOM models were FDR (BH method) corrected, and a significant sub-hypothesis test at a level of *adj.P* < 0.05 was counted towards the *W*-value. “***” = ≥ *W*_0.9_, “**” = ≥ *W*_0.8_, “*” = ≥ *W*_0.7_, “.” = ≥ *W*_0.6_. The full list of the differential ASV abundance is available in Supplemental Table 6. Corresponding differential genera abundance has been depicted in Supplemental Fig. 3, and the full list is available in Supplemental Table 7. **B** Venn diagram showing the common differential ASV abundance among offspring from the different combinations of reciprocal crosses as indicated. **C** Relative abundance of the differential ASV in the genus *Faecalibaculum* lineage among different reciprocal crosses. **D** Weighted UniFrac *β*-diversity principal coordinate plot of the offspring gut microbiota stratified by diets. The colors represent reciprocal cross pairs as indicated. The ellipse indicates the 95% CI of the clusters according to the reciprocal cross

To elucidate the parental-origin (PO) effect on offspring gut microbiota, we compared *β*-diversity between reciprocal cross pairs within each diet group. PO explained approximately 20 to 58% of the *β*-diversity variability, depending on the RIX pairs and diet groups (Supplemental Table 5). Figure 5D presents the PCoA plots for weighted UniFrac *β*-diversity, comparing RIX cross pairs exposed to different in utero diets. These findings suggest that the PO may modulate the associations between bacterial composition and diet during development. Consequently, we conducted ANCOM-2 between the crosses in a RIX pair at the ASV (Fig. 5A, B and Supplemental Table 12) and genus levels (Supplemental Table 13) stratified by diet (e.g., CC001xCC011 vs. CC011xCC001 in CON) to identify specific bacteria influenced by the PO. We identified several bacterial ASVs that were differentially abundant between offspring from reciprocal crosses within different treatment groups. For example, abundance of an ASV in the *Akkermansia* lineage was 2.66-fold higher in CC011xCC001 offspring in the control group than in CC001xCC011 offspring in the same diet (Supplemental Table 12). Similarly, several ASVs belonging to the genus* Muribaculaceae* were found to be more common in CC001xCC011 offspring in the control, LP, and LVD groups than in the corresponding CC011xCC001 offspring. Figure 5B shows the overlap of the differentially abundant ASVs between the different reciprocal crosses within the control group. Among the several differentially abundant ASVs, we identified an ASV belonging to the genus *Faecalibaculum* (Fig. 5C) and an ASV belonging to *Lachnospiraceae_FCS020_group* that are common among all three reciprocal crosses, indicating a consistent PO effect on the abundance of *Faecalibaculum* and *Lachnospiraceae_FCS020_group* across different mouse strains in this study.

### Gut microbial composition associated with bodyweight in adulthood

**Fig. 6:**
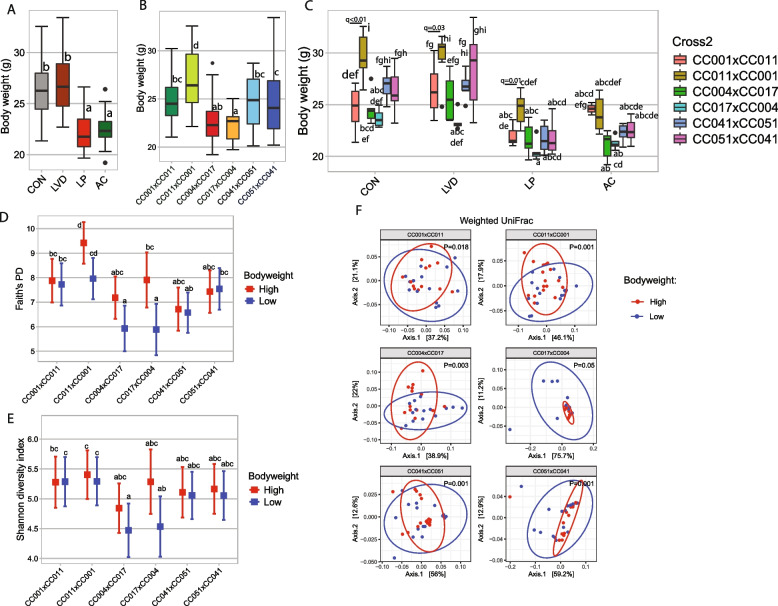
Gut microbial diversity is associated with bodyweight across all reciprocal crosses. Distribution of bodyweight among **A** treatment groups, **B** crosses, and **C** reciprocal cross with each diet. Boxes without a common letter are significantly different from others. *P*-values were corrected for multiple comparisons using the Benjamini–Hochberg method, and adjusted *P*-values (*q*-values) were reported. Comparisons of **D** Faith’s PD *α*-diversity and **E** Shannon diversity index between high and low bodyweight for each cross. **F** Comparisons of weighted UniFrac *β*-diversity between high and low bodyweight in each cross

The microbiome is one of the many factors affecting bodyweight and adiposity. For example, bodyweight varied across the four diet treatments (Fig. 6A) and between reciprocal crosses (Fig. 6B), indicating distinct environmental and genetic factors contributing to bodyweight. ANCOVA analysis revealed that independent of genetic background, antibiotic exposure during the developmental period significantly (*P* < 0.0001) reduced offspring bodyweight at 8 weeks of age by 14.8% (Fig. 6A). We also observed a significant interaction (*P* = 0.044) between genetic background and AC diet treatment on bodyweight. Further analysis revealed that CC011xCC001, CC041xCC051, and CC051xCC041 offspring in the AC group had significantly lower bodyweight than those in the corresponding control groups (Fig. 6C). To further determine the effect of PO on bodyweight, we compared bodyweight between crosses within a RIX pair (e.g., CC001xCC011 vs CC011xCC001) via the Wilcoxon test (Table 2). We found significant differences in bodyweight among the reciprocal cross pairs in the control, LP, and LVD diet groups. As shown previously [19], CON-treated CC011xCC001 offspring weighed more than their reciprocal cross (CC001xCC011), but this effect was ameliorated by AC treatment (Fig. 6C). The other two sets of reciprocal crosses showed no difference in bodyweight under either dietary treatment. This result highlights the long-term effects of maternal antibiotic exposure on offspring bodyweight which differ by genetic background and PO. Similar to the AC diet, we found that the LP diet also significantly (*P* < 0.001) reduced offspring bodyweight by 15% at 8 weeks of age, independent of genetic background, indicating long-term growth retardation (Fig. 6A). Additionally, we observed a significant (*P* < 0.01) interaction effect between diet and cross. The post hoc analysis revealed that the offspring from CC011xCC001, CC041xCC051, and CC051xCC041 crosses fed the LP diet had significantly lower bodyweight compared to their control diet group (Table 2 and Fig. 6C), which was not observed in offspring from the CC001xCC011, CC004xCC017, and CC017xCC004 crosses. We further determined whether PO modulated the effect of protein deficiency during the developmental period on offspring growth between crosses within each RIX pair in the LP group. We found that offspring from CC011xCC001 had a significantly eater bodyweight than those from its reciprocal cross CC001xCC011 in the LP diet (*P* < 0.05, *q* = 0.028) (Fig. 6C and Table 2). In contrast to protein deficiency, maternal vitamin D deficiency did not have a significant (*P* = 0.07) effect on offspring bodyweight at 8 weeks of age (Fig. 6A), nor did we observe any significant (*P* = 0.61) diet by cross interactions. However, similar to the other diet perturbations, the offspring from CC011xCC001 had a significantly greater bodyweight compared to its reciprocal cross CC001xCC011 (*P* < 0.001, *q* = 0.012) (Fig. 6C and Table 2) in the LVD groups. Offspring from CC004xCC017 and CC017xCC004 also had different bodyweight in the LVD group but were not robust to corrections for multiple comparisons (*P* = 0.032, *q* = 0.25).

**Fig. 7:**
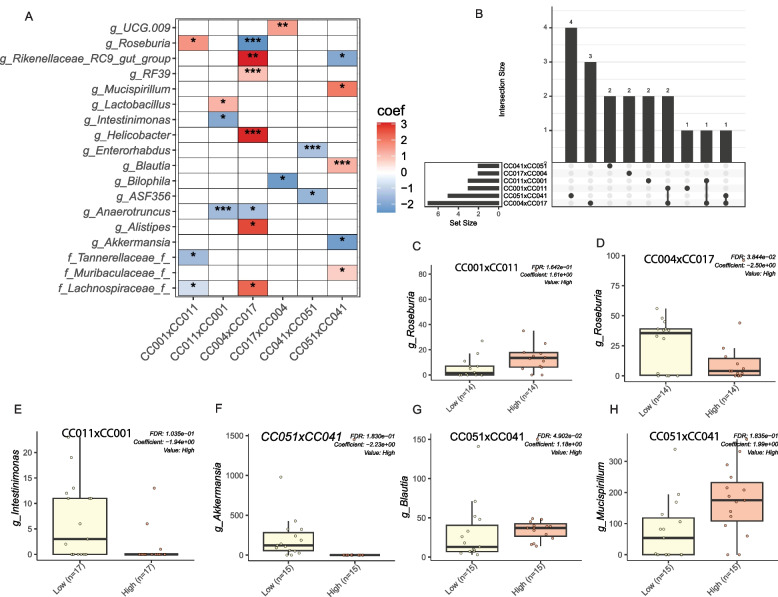
Specific gut bacteria are associated with bodyweight across all CC mouse crosses. **A** Heatmap showing the association between bacterial genus and bodyweight. The top 20 most influential (based on cumulative absolute coefficient value) genera were selected for the graph. On the *Y*-axis, maximum taxonomic information has been presented. The association was determined by MaAsLin-2. Bodyweight was categorized as high (above median) and low (below median) for each of the CC crosses. MAaslin-2 model was adjusted for diet. Red indicates a positive association between gut genera abundance and bodyweight, whereas blue indicates a negative association between gut genera abundance and bodyweight. **B** Upset plot to showcase the common gut genera found associated with bodyweight among different CC crosses. **C**, **D**, **E**, **F**, **G**, **H** indicating the abundance of *Roseburia*, *Intestinimonas*, *Akkermansia*, *Blautia*, and *Mucispirillum* in CC mice strains having high and low bodyweight as indicated

Finally, we sought to identify whether common microbial diversity and abundance were associated with obesity across the various F1 crosses. To do so, we dichotomized bodyweight within a reciprocal cross and examined the association of bodyweight with microbial *α*-diversity after adjusting for dietary treatments (Fig. 6 D, E, Supplemental Fig. 4, and Supplemental Table 14). Overall, there were subtle differences by F1 cross. Only CC017xCC004 had a significantly higher Faith’s PD in the higher bodyweight group than in the lower bodyweight group (Fig. 6D and Supplemental Table 14). Similar to *α*-diversity analysis, we determined the association between gut microbial *β*-diversity and bodyweight using ADONIS upon adjustment for diet and found that 4 to 16% variability of the bodyweight depending on the cross and diversity matrix (Fig. 6F and Supplemental Table 15). To better quantify the relationship between specific bacterial genera and bodyweight, we employed MaAsLin-2 and identified specific genera that were differentially abundant between high and low bodyweight in different crosses (Fig. 7A and Supplemental Table 16). Several of these associations were replicated in different crosses (Fig. 7B), but the majority of the associated genera were specific to an individual cross. A number of the genera are known to be associated with bodyweight (Fig. 7C, D, E, F, G, H) and include *Roseburia* which is highly abundant when bodyweight increases in CC001xCC011. The F1 cross CC051xCC041 identified a high abundance of *Akkermansia* in lower weight mice and a high abundance of *Blautia* and *Mucispirillum* in higher weight  CC051xCC041 mice. Similar to genus level, significant associations between bacterial ASV and bodyweight was also observed (Supplemental Fig. 5 and Supplemental Table 17).

## Discussion

Environmental insults during the developmental period can increase susceptibility to diseases later in life [5, 6, 15, 16]. These perturbations can be partially mediated through dysbiosis [7, 12, 13]. Although few studies have evaluated the effects of a prenatal diet on the gut microbiota of progeny mice, these studies are limited to a high-fat diet or dietary fiber [38]. Studies evaluating the effects of environmental insults such as antibiotic use, protein deficiency, or vitamin D deficiency during the developmental period on offspring gut microbiota are very limited, and no studies have evaluated the longer-term effects. In this study, we determined whether maternal consumption of an AC, LP, or LVD diet during gestation through weaning affects offspring gut microbiota and growth at 8 weeks of age. To mimic human undernutrition, our study dams were fed experimental diets 5 weeks prior to pregnancy, through gestation, until weaning. The F1 mice were generated from multiple reciprocal crosses of genetically advanced multiparental CC mice, which gave us the unique opportunity to evaluate the parent-of-origin (PO) effect and the broad effect of genetics on offspring gut microbiota and growth. Our study has several novel findings, which are discussed below.

Antibiotics have an overwhelming effect on gut microbial diversity and composition [39], which may contribute to an increased risk of various diseases in offspring [15, 16]. The altered metabolism and body composition due to neonatal antibiotic exposure can be transferred to healthy animals by fecal microbiota transplantation, indicating that the altered microbiota is responsible for the phenotypic variation [1]. However, our understanding of the effects of maternal antibiotic use on offspring gut microbiota and health is very limited. In this study, we evaluated whether maternal antibiotic exposure (1% succinyl sulfathiazole) during development has any effect on offspring gut microbiota and bodyweight at 8 weeks of age. We found that compared with the control, maternal antibiotic use during the developmental period significantly decreased offspring gut microbial diversity at 8 weeks of age. In support of our findings, a previous study reported that maternal antibiotic use during birth reduced microbiota richness in 3-month-old infants [40]. Other studies also reported that the administration of perinatal antibiotics [41] or antibiotics to newborns [42] reduces microbial diversity in 2-month-old infants. A rodent study reported that even a transient antibiotic-induced perturbation of the neonatal gut microbiota resulted in a permanent alteration in metabolism, immunity, and body composition [1]. In our study, we observed reduced microbial diversity and significantly lower bodyweight in offspring from the AC diet group compared to controls. The lower bodyweight in the current study differs from large epidemiological studies [43] and could be due to reduced folate availability as succinyl sulfathiazole has been used in studies of folate deficiency [44]. This further suggests that the long-term effects of antibiotic exposure on bodyweight in adults are likely due to gut microbiota alterations.

Similar to antibiotic treatment, protein deficiency during the developmental period significantly reduced offspring bodyweight at 8 weeks of age, indicating growth retardation. Research indicates that a low-protein diet reduces the intestinal villus-crypt ratio in comparison to a balanced, nutritionally adequate diet [45]. This diminished intestinal villus-crypt ratio may result in compromised nutrient absorption and ultimately suboptimal growth. In line with our study, recent studies have demonstrated that a protein-deficient diet causes weight loss [46] and influences the gut microbiota [47]. Studies have shown that the composition and function of the intestinal microbiota may contribute to the onset of Kwashiorkor, a disorder believed to be prevalent among children who consume a low-protein diet [7]. Another study reported that maternal animal protein consumption during pregnancy was associated with neonatal first-pass microbiota [48], indicating that maternal protein status or source of protein may directly modulate early-life microbiota in offspring. Therefore, we also assayed the gut microbiota of offspring during adulthood and found that the LP diet and genetics significantly affected both *α*-diversity and *β*-diversity. In particular, the *α*-diversity of CC011xCC001 offspring in the LP group was significantly lower than that of CC011xCC001 offspring in the AIN-93G group, indicating that the effect of protein deficiency during the developmental period on the gut microbiota may be dependent on genetic background. Since CC011xCC001 offspring in the low-protein group also had lower bodyweight, the growth retardation might be partially explained by the altered gut microbiota. Consistent with our results, a recent mouse study [46] revealed that mice fed a low-protein diet had lower bodyweight and altered microbial diversity at 4 weeks of age. We also observed lower bodyweight in offspring from CC041xCC051 and CC051xCC041 crosses in the LP group than in the control; however, these offspring did not have any difference in microbial diversity compared with their corresponding controls, which indicates that protein deficiency during the developmental period may result in diminished growth at 8 weeks both through the gut microbiota and via non-microbiota pathways.

Our study revealed that compared with the control, protein deficiency during the developmental period resulted in a lower abundance of several bacteria, including *Bifidobacterium* and *Akkermansia*, at 8 weeks of age, depending on the genetic background. Importantly, *Akkermansia* is a mucin-degrading bacterium that can obtain amino acids from the host’s glycoprotein mucins [49]. Therefore, the reduced abundance of *Akkermansia* and *Bifidobacterium* observed in our study could be due to altered intestinal structure or function, which was previously reported [45]. A recent study [46] showed that low-protein diet treatment in adult mice increased the abundance of the Streptococcaceae and Clostridiales families. Similarly, another study reported altered Verrucomicrobia (predominantly *Akkermansia*) abundance due to protein restriction [50]. To the best of our knowledge, our report is the first to demonstrate that protein deficiency during the developmental period affects offspring growth and the gut microbiota during adulthood, which is modulated by genetic background.

Maternal vitamin D status affects vitamin D availability to offspring, which can result in diminished development during gestation and lactation [51, 52]. In addition to immune cells, vitamin D receptors (VDRs) are found on intestinal enterocytes, where they act as transcription factors for the secretion of antimicrobial peptides such as cathelicidin and *β*-defensin, which shift the intestinal microbiota towards a healthier composition [53, 54]. Therefore, we determined the effects of vitamin D deficiency during development on offspring bodyweight and the gut microbiota later in life. In contrast to our hypothesis, maternal vitamin D deficiency did not significantly alter bodyweight or microbial *α*-diversity. Consistent with our findings, vitamin D supplementation during pregnancy did not alter infant gut microbial *α*-diversity in Denmark [55] or the USA [56] within 1 year and 6 months of age, respectively. Similarly, in piglets, maternal vitamin D supplementation during gestation and lactation did not affect *α*-diversity compared with the control at 3 weeks of age [57]. These results show that vitamin D deficiency during development might have no effect or, at most, a transient effect on the gut microbiome, which may recover over time.

The link between host genetics and the gut microbiome is well defined [58]. The use of CC-RIX strains provides a unique opportunity to investigate the parent-of-origin (PO) effects on the gut microbiota and health of offspring. Since F1 offspring of reciprocal cross pairs are genetically identical except for the sex chromosomes and mitochondrial genome, PO effects may be driven by these genetic differences among the offspring, by maternal uterine or placental effects driven by differences in maternal genetic background, or by epigenetic phenomena such as genomic imprinting effects [23] that differ among the genetic backgrounds. Our study found that the gut microbiota and bodyweight vary depending on the progeny genetic background and PO, which was replicated in our previous study [19]. For example, we observed that the bodyweight of CC011xCC001 offspring fed the control, LVD, or LP diet significantly differed from that of their reciprocal cross CC001xCC011 fed a corresponding diet. However, we did not observe this PO-dependent growth alteration in the AC diet, indicating that the effect of PO on growth in CC001xCC011 and CC011xCC001 was mediated through the gut microbiota. Indeed, we observed differences in microbial diversity and composition between CC011xCC001 and CC001xCC011 offspring in the control, LVD, and LP diets. To the best of our knowledge, our study is the first to report the effects of PO on offspring gut microbiota and bodyweight during adulthood. Among different gut bacteria, we found that the abundance of an ASV in the genus *Faecalibaculum* is highly influenced by PO. *Faecalibaculum* is associated with several biological functions, including those that are beneficial for ulcerative colitis, inflammatory bowel diseases, diabetes, and colorectal cancer [59]. A recent study [60] reported that *Faecalibaculum* was enriched in male but not in female high-fat diet-fed mothers. Furthermore, *Faecalibaculum* produces the short-chain fatty acid butyrate at an effective biological concentration [61] sufficient for inhibiting histone deacetylase (HDAC) activity [62]. Thus, the differential abundance of *Faecalibaculum* that we observed in our study could have important impacts on disease susceptibility or gut function.

There is considerable interest in the role of microbiota in bodyweight and obesity, given its critical role in digestion and energy harvesting within the gut. Generally, there is reduced bacterial diversity in obese subjects [63] and changes in specific organisms when animal models undergo a diet-induced obesity regime [64]. Additionally, complex genetic interactions regulate the microbiome composition [65]. Given the complexity of the many factors influencing bodyweight, we performed an exploratory analysis to identify whether the microbiota is associated with bodyweight after controlling for diet. Our experimental design was complicated but allowed for an exploratory analysis to identify differential abundance of microbial genera and ASV associated with bodyweight. We hypothesized that we could observe a subset of “core” microbiota that were associated despite dietary and genetic perturbations. Indeed, there were a number of genera and ASV associated with bodyweight but robust consistent signals within a reciprocal cross were muted. Although a core group of microbiota did not emerge, we observed that a number of microbiota, such as *Roseburia*, *Intestinimonas*, *Akkermansia*, *Blautia*, and *Mucispirillum*, were significantly associated with bodyweight.

Our study presents several novel findings, though some aspects merit further investigation. We did not measure the gut microbiota of the mother or offspring during early life. However, several studies have reported altered gut microbiome composition after protein or vitamin D deficiency in adults [46, 66, 67]. Furthermore, our AC diet contains more choline bitartrate (a methyl donor) than the control diet does, which may also modulate the abundance of some gut bacteria [68]. Additionally, mice are known to be coprophagic, so we cannot rule out the pup’s exposure to unabsorbed antibiotics via coprophagy. Moreover, our study did not include female mice; thus, we cannot generalize the PO results for both male and female offspring. Finally, our extraction protocol for this study did not utilize mechanical bead beating which may have limited our ability to detect some bacteria [69]. Further studies are needed to evaluate the effect of sex-developmental and/or environment-genetic interactions on the colonization of the gut microbiota and its eventual role in physiology and disease susceptibility later in life.

## Conclusions

In conclusion, our study demonstrated that antibiotic use, protein deficiency, or vitamin D deficiency during the developmental period has long-lasting effects on offspring gut microbiota and growth, which are further modulated by maternal and offspring genetic background in a PO-dependent manner. Our study suggests the importance of reducing antibiotic use, increasing protein balance, and improving maternal vitamin D status during pregnancy and lactation to improve offspring health outcomes through a balanced gut microbiota.

## Supplementary Information

Additional file 1: Supplemental Figure 1: (A) Mean relative abundance of the top 20 genera in offspring from different reciprocal crosses grouped by maternal prenatal diet. (B) Mean relative phylum abundance in offspring from different reciprocal crosses grouped by different maternal prenatal diets.

Additional file 2: Supplemental Figure 2: (A) Unweighted UniFrac and (B) Bray-Curtis β-diversity principal coordinate plots of the offspring gut microbiota by reciprocal crosses. The different colors represent different maternal diets as indicated. The ellipse indicates the 95% CI of the clusters by maternal diet.

Additional file 3: Supplemental Figure 3: Differential gut microbiota abundance between the AC and control diets. Volcano plot of the differential ASV abundance in CC011xCC001 offspring from the AC and control groups determined by ANCOM-2 analysis. A structural zero represents the presence of bacteria in one group and their complete absence in another group.

Additional file 4: Supplemental Figure 4: Comparisons of observed ASV between high and low bodyweight.

Additional file 5: Supplemental Figure 5: Gut bacterial ASV is associated with bodyweight across all CC mouse crosses. (A) Heatmap of showing association between bacterial ASV and bodyweight. The top 20 most influential (based on cumulative absolute coefficient value) ASV were selected for the graph. On the Y-axis, maximum taxonomic information has been presented. The association was determined by MAaslin-2. Bodyweight was categorized high (above median) and low (below median) for each of the CC cross . MAaslin-2 model was adjusted for diet. Red indicates positive association between gut genera abundance and bodyweight whereas blue indicates negative association between gut genera abundance and bodyweight. (B) Upset plot to showcase the common gut genera found associated with bodyweight among different CC cross . (C-H) indicating the abundance of ASVs from Muribaculaceae, Bacteroides, and Oscillospiraceae abundance in CC mice strains having high and low bodyweight as indicated.

Additional file 6: Supplemental Tables 1 through 13.

## Data Availability

The datasets generated and/or analyzed during the current study are available in the NCBI Sequence Read Archive (SRA) database under the BioProject ID “PRJNA1184353”.

## Abbreviations

ASVs: Amplicon sequence variants
AC: Antibiotic-containing
CC: Collaborative Cross
CON: Control
FDR: False discovery rate
GI: Gastrointestinal
LP: Low protein
LVD: Low vitamin D
PO: Parent-of-origin
PCoA: Principal coordinate
QIIME2: Quantitative Insights into Microbial Ecology Version 2
RIX: Recombinant inbred intercross
SRA: Sequence Read Archive
VDRs: Vitamin D receptors
