# Supplementary figures and images for: The impact of early-life exposures on growth and adult gut microbiome composition is dependent on genetic strain and parent- of- origin
Source: Microbiome. 2025 Jun 16;13:143. doi: 10.1186/s40168-025-02130-w (PMC12168266; doi:10.1186/s40168-025-02130-w)

A

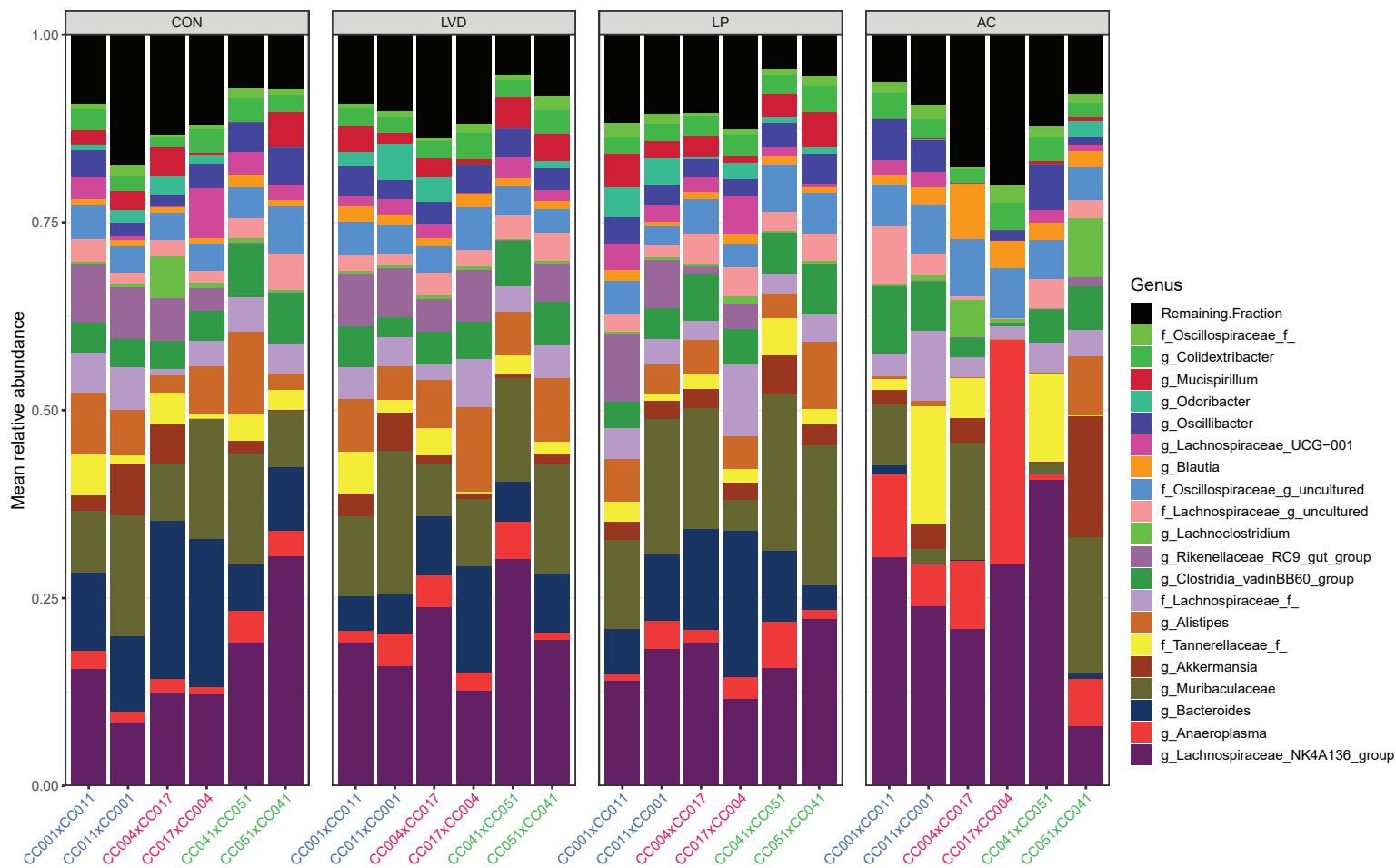

B

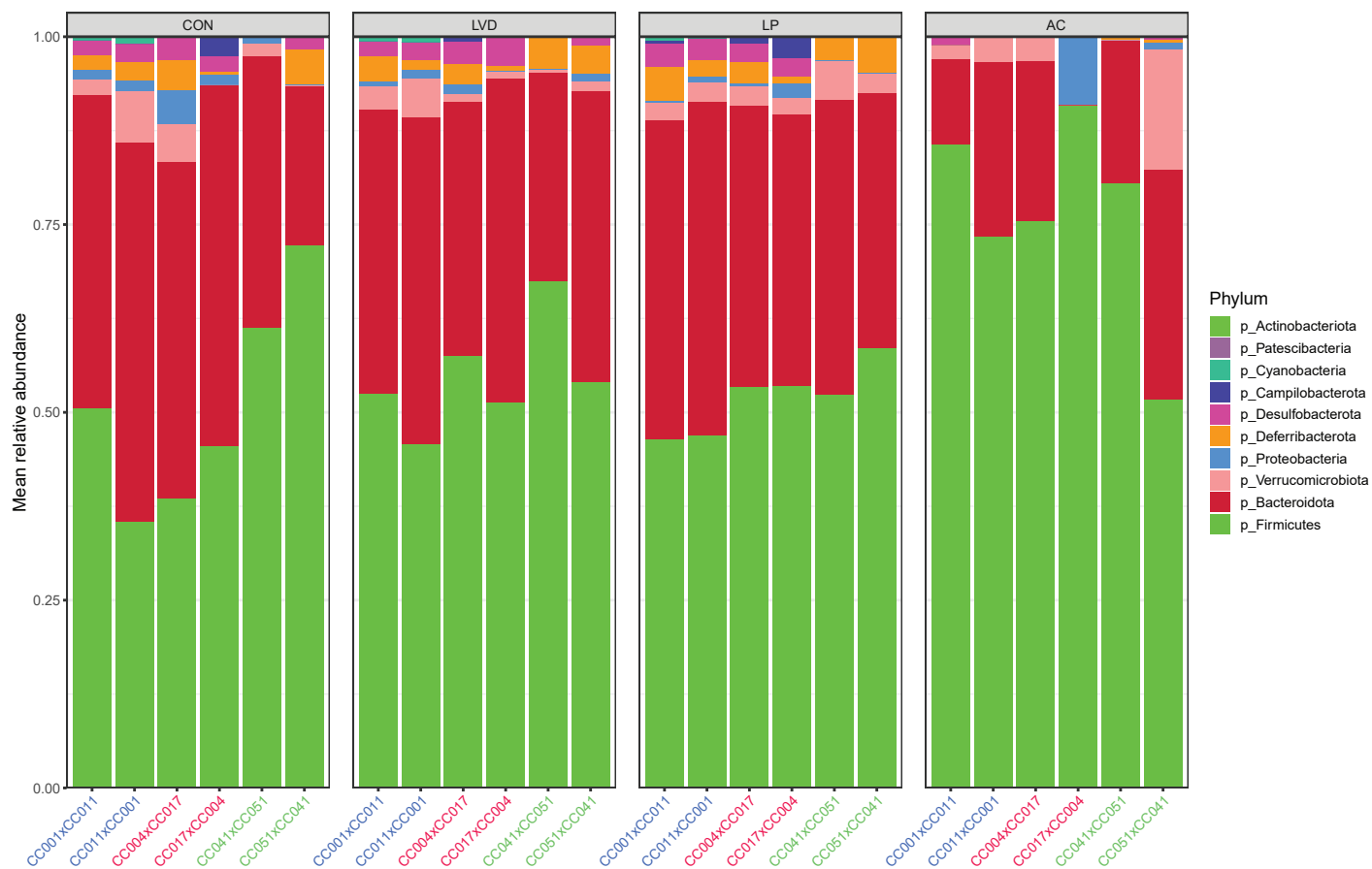

Supplement: Supplementary file 2 — Additional file 1: Supplemental Figure 1: (A) Mean relative abundance of the top 20 genera in offspring from different reciprocal crosses grouped by maternal prenatal diet. (B) Mean relative phylum abundance in offspring from different reciprocal crosses grouped by different maternal prenatal diets. [file 40168_2025_2130_MOESM1_ESM.pdf]

A

## Unweighted UniFrac

AC vs. CON

LP vs. CON

LVD vs. CON

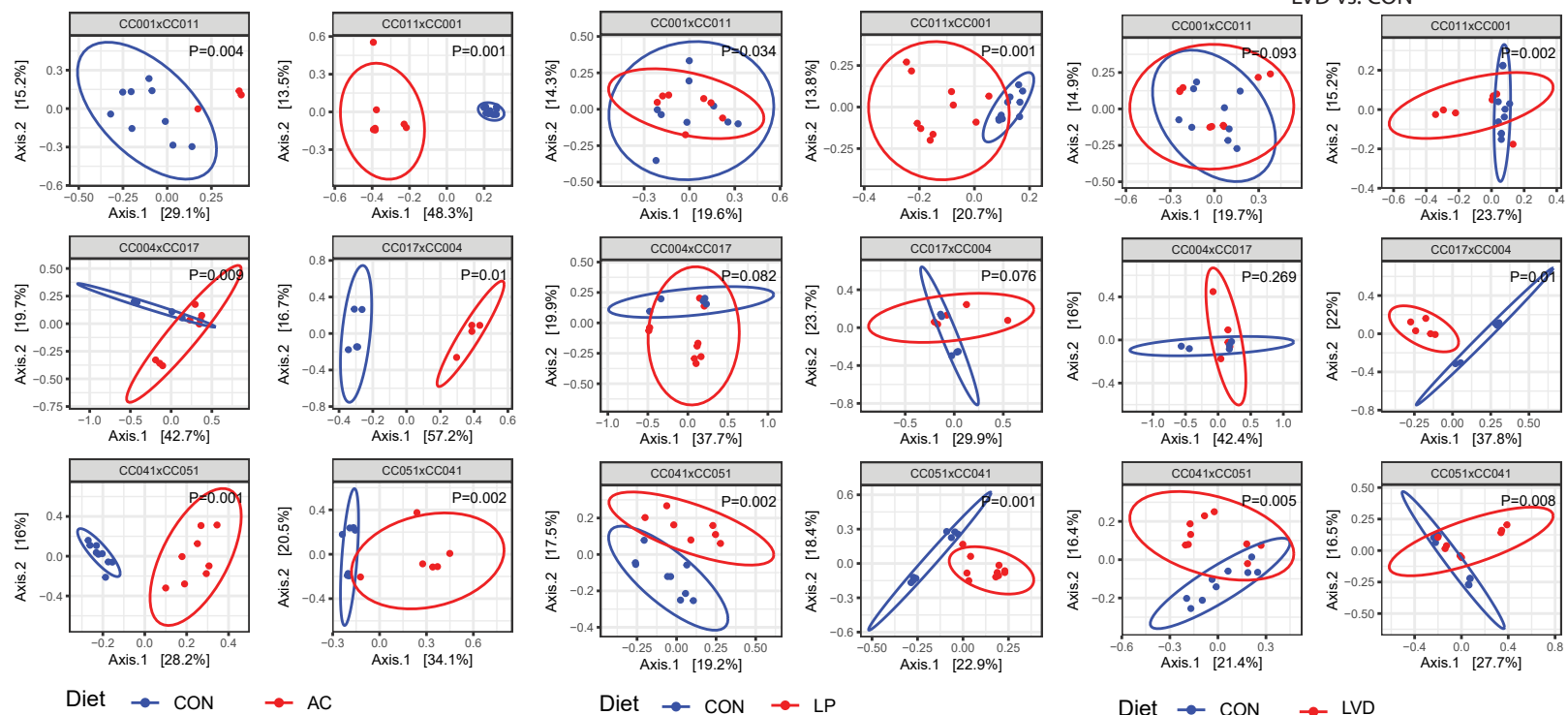

B

## Bray-Curtis

AC vs. CON

LP vs. CON

LVD vs. CON

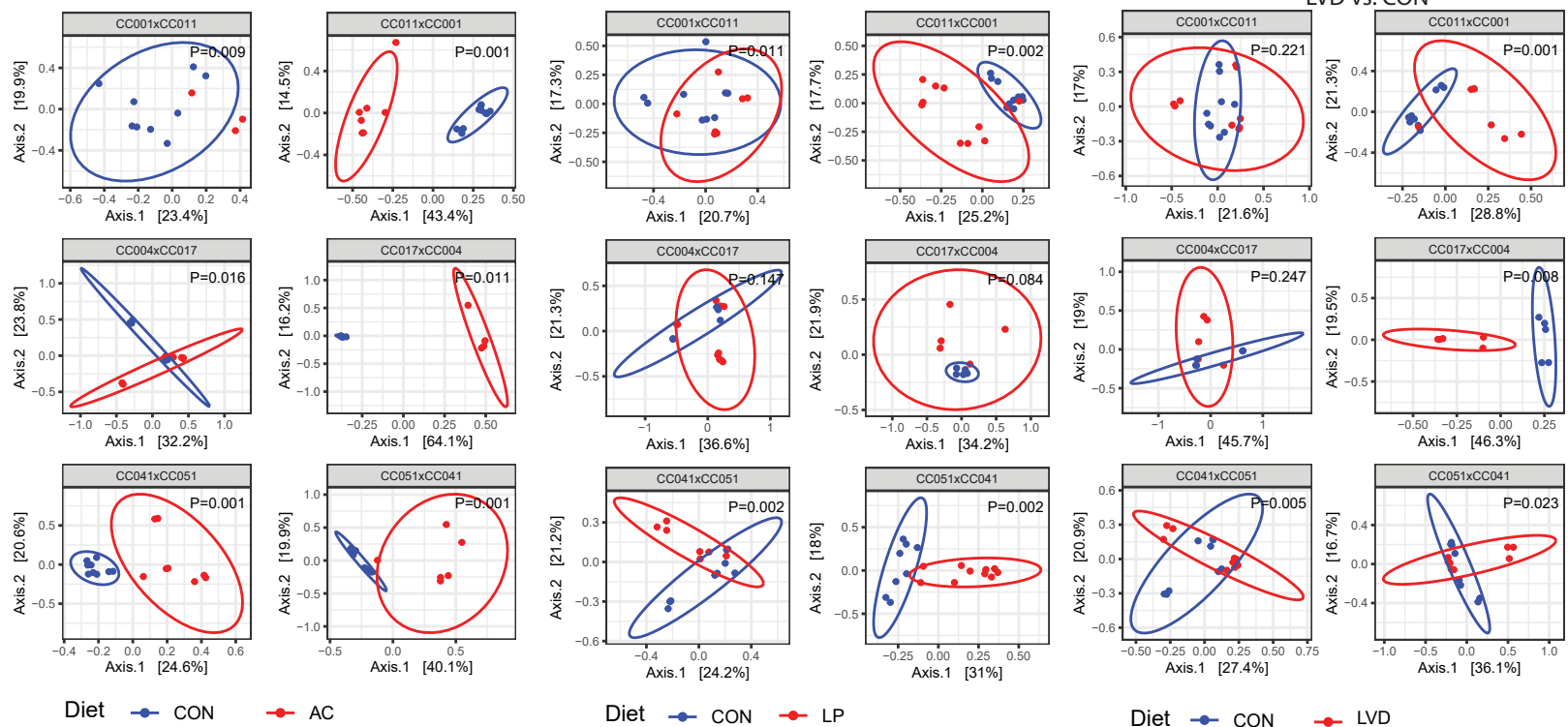

Supplement: Supplementary file 3 — Additional file 2: Supplemental Figure 2: (A) Unweighted UniFrac and (B) Bray-Curtis β-diversity principal coordinate plots of the offspring gut microbiota by reciprocal crosses. The different colors represent different maternal diets as indicated. The ellipse indicates the 95% CI of the clusters by maternal diet. [file 40168_2025_2130_MOESM2_ESM.pdf]

W statistic

CLR mean difference

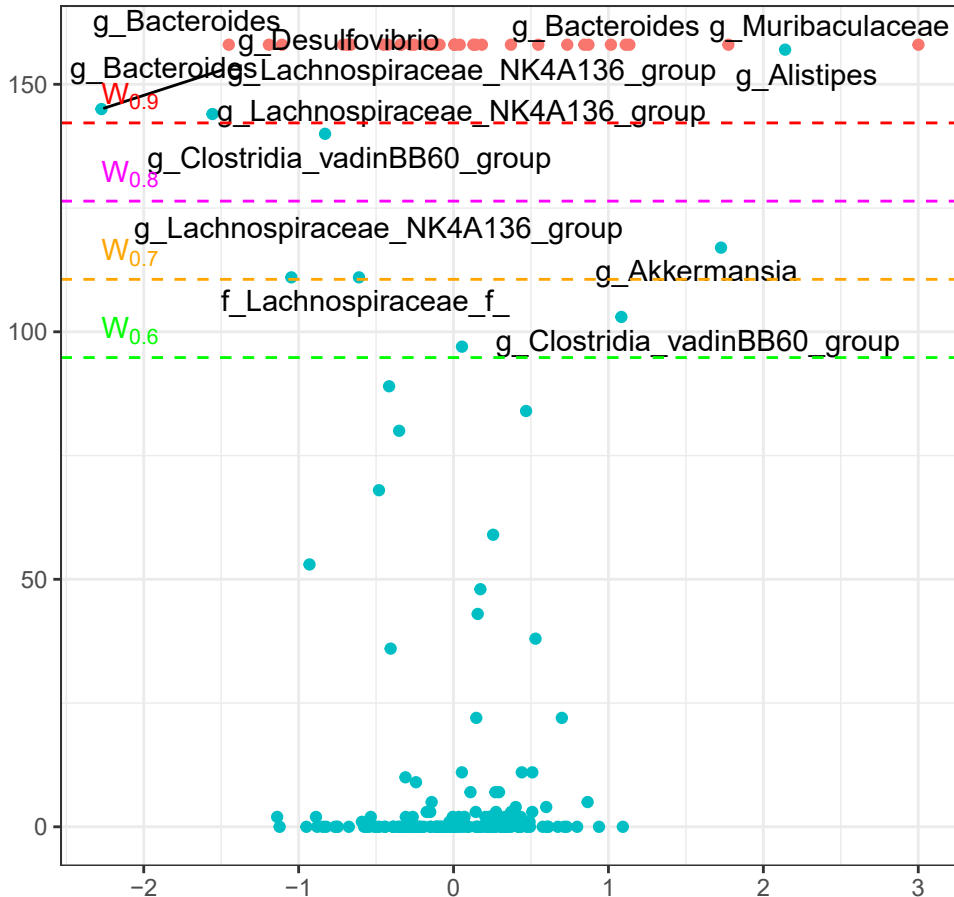

Structural zero

- Yes
- No

Supplement: Supplementary file 4 — Additional file 3: Supplemental Figure 3: Differential gut microbiota abundance between the AC and control diets. Volcano plot of the differential ASV abundance in CC011xCC001 offspring from the AC and control groups determined by ANCOM-2 analysis. A structural zero represents the presence of bacteria in one group and their complete absence in another group. [file 40168_2025_2130_MOESM3_ESM.pdf]

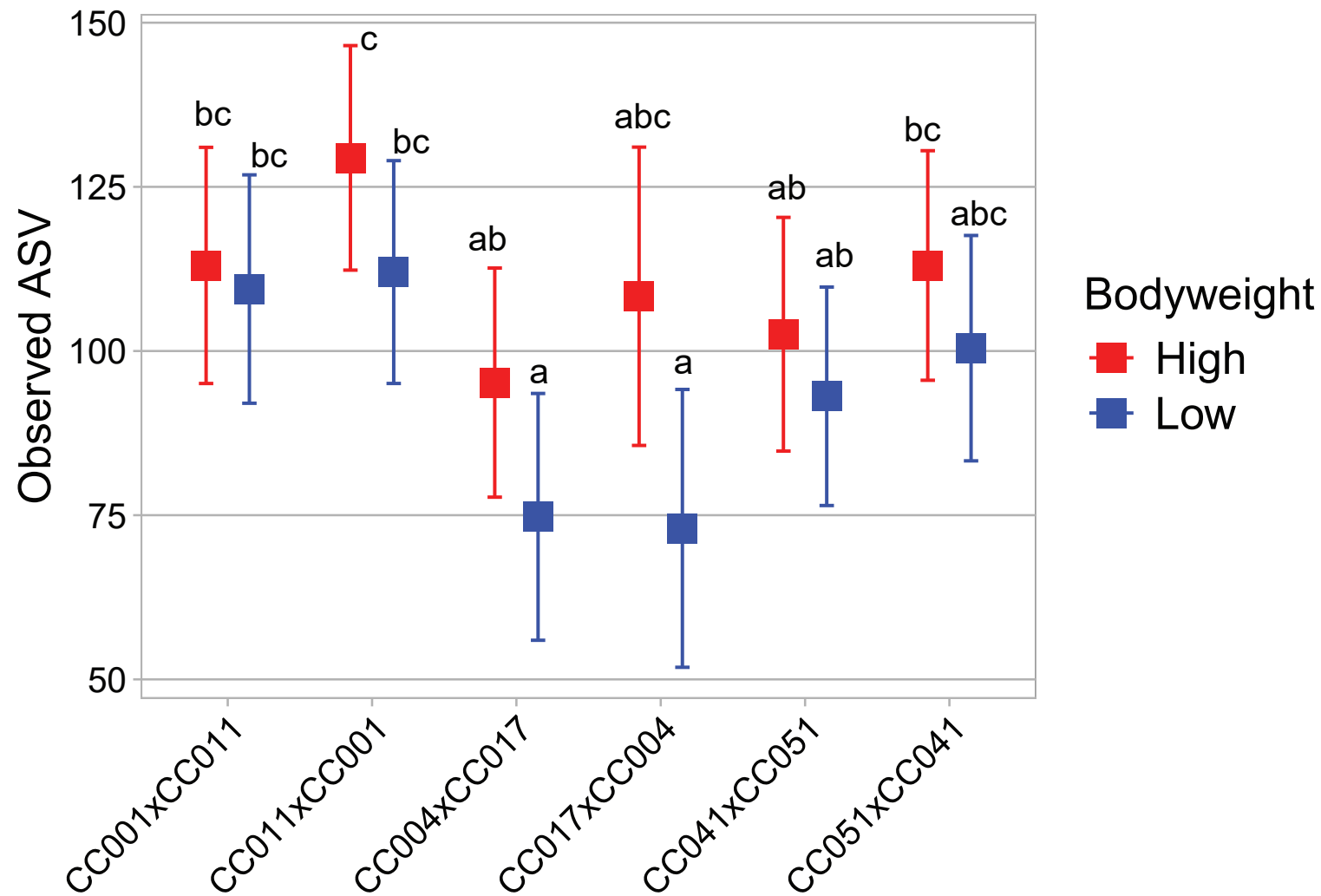

Supplement: Supplementary file 5 — Additional file 4: Supplemental Figure 4: Comparisons of observed ASV between high and low bodyweight. [file 40168_2025_2130_MOESM4_ESM.pdf]

A

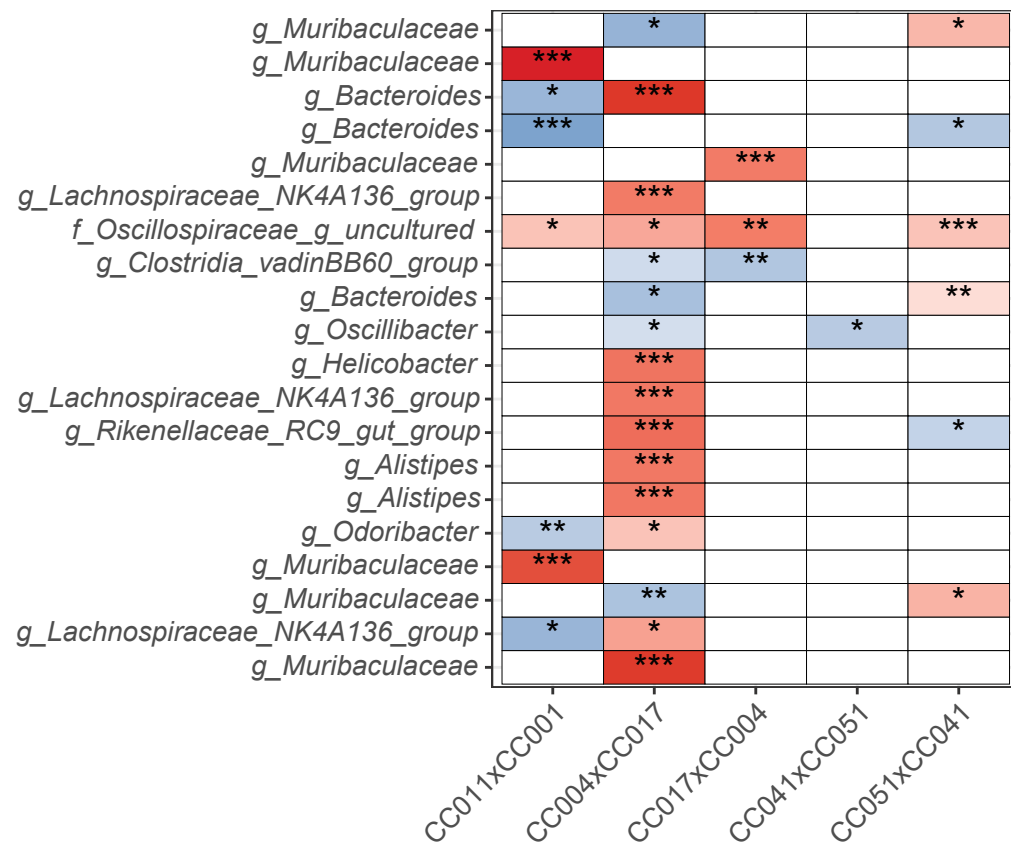

B

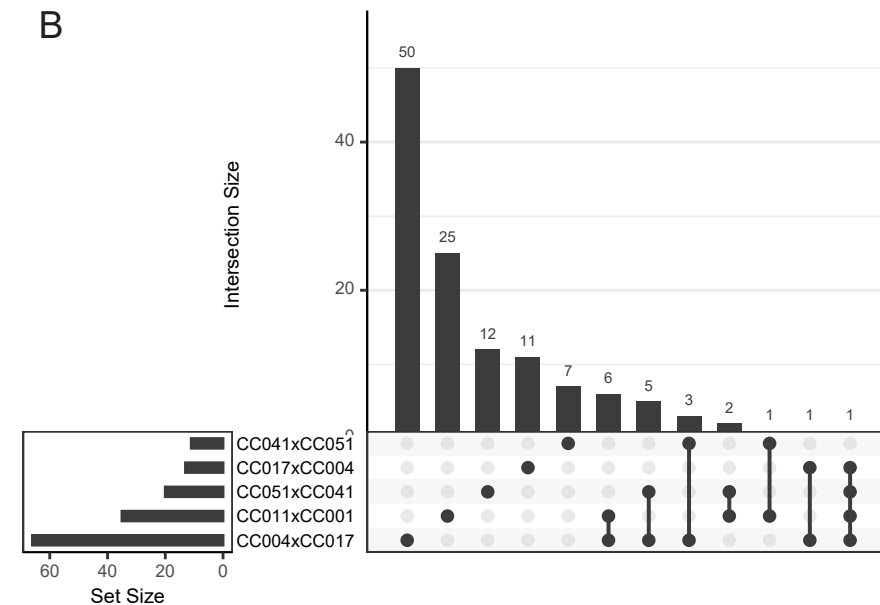

C

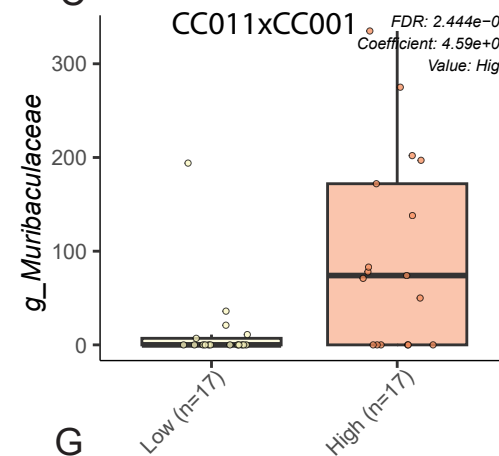

D

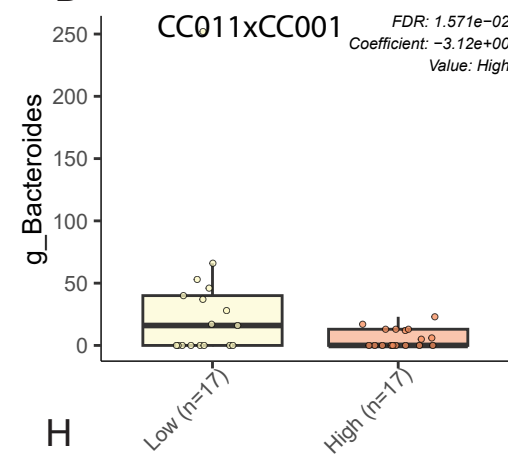

E

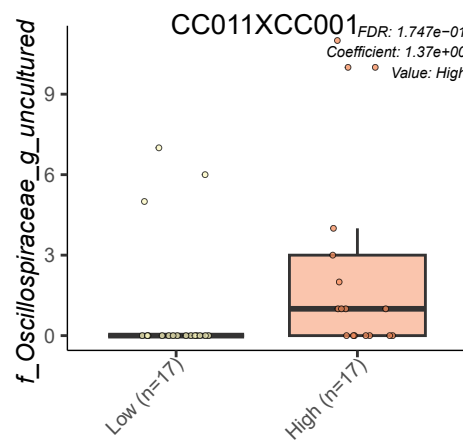

F

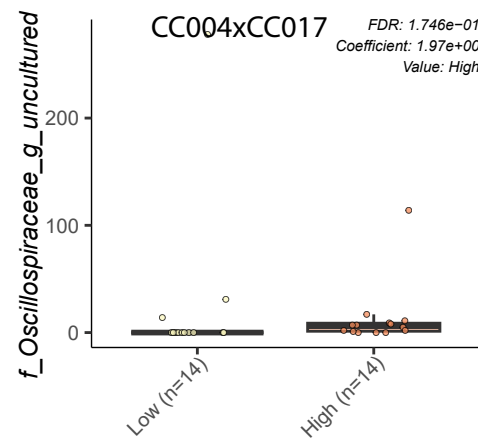

G

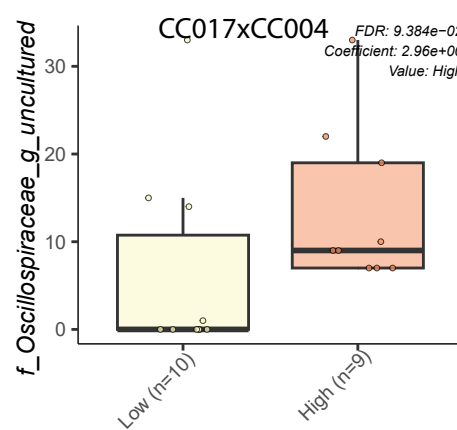

H

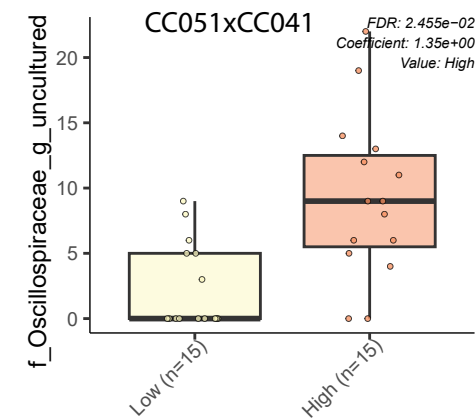

Supplement: Supplementary file 6 — Additional file 5: Supplemental Figure 5: Gut bacterial ASV is associated with bodyweight across all CC mouse crosses. (A) Heatmap of showing association between bacterial ASV and bodyweight. The top 20 most influential (based on cumulative absolute coefficient value) ASV were selected for the graph. On the Y-axis, maximum taxonomic information has been presented. The association was determined by MAaslin-2. Bodyweight was categorized high (above median) and low (below median) for each of the CC cross . MAaslin-2 model was adjusted for diet. Red indicates positive association between gut genera abundance and bodyweight whereas blue indicates negative association between gut genera abundance and bodyweight. (B) Upset plot to showcase the common gut genera found associated with bodyweight among different CC cross . (C-H) indicating the abundance of ASVs from Muribaculaceae, Bacteroides, and Oscillospiraceae abundance in CC mice strains having high and low bodyweight as indicated. [file 40168_2025_2130_MOESM5_ESM.pdf]
